# Lactobacilli Isolated From Wild Boar (*Sus scrofa*) Antagonize *Mycobacterium bovis* Bacille Calmette-Guerin (BCG) in a Species-Dependent Manner

**DOI:** 10.3389/fmicb.2019.01663

**Published:** 2019-07-30

**Authors:** Maria Bravo, Theo Combes, Fernando O Martinez, Rosario Cerrato, Joaquín Rey, Waldo Garcia-Jimenez, Pedro Fernandez-Llario, David Risco, Jorge Gutierrez-Merino

**Affiliations:** ^1^Innovación en Gestión y Conservación de Ungulados SL, Cáceres, Spain; ^2^Departamento de Sanidad Animal, Facultad de Veterinaria, Universidad de Extremadura, Cáceres, Spain; ^3^Department of Biochemical Sciences, School of Biosciences and Medicine, University of Surrey, Guildford, United Kingdom; ^4^Department of Nutritional Sciences, School of Biosciences and Medicine, University of Surrey, Guildford, United Kingdom

**Keywords:** antagonism, Bacillus Calmette-Guerin, bovine tuberculosis, lactobacilli, *Mycobacterium bovis*, probiotics, wild boar

## Abstract

**Background**: Wildlife poses a significant burden for the complete eradication of bovine tuberculosis (bTB). In particular, wild boar (*Sus scrofa*) is one of the most important reservoirs of *Mycobacterium bovis*, the causal agent of bTB. Wild boar can display from mild TB lesions, usually found in head lymph nodes, to generalized TB lesions distributed in different anatomical regions; but rarely clinical signs, which complicates the diagnosis of *Mycobacterium bovis* infection and bTB control. Among the possibilities for this variability in lesion distribution is the influence of the host-beneficial commensal-primed immune barrier. In this respect, beneficial microbes may delay bTB dissemination as a consequence of an antagonistic competition for nutrients and phagocytes. In order to explore this possibility, we have tested whether typical commensals such as lactobacilli have the capacity to reduce the survival rate of the surrogate *M. bovis* strain Bacillus Calmette-Guerin (BCG); and to modulate its phagocyte intake.

**Results**: Three *Lactobacillus* species, *L. casei*, *L. plantarum,* and *L. salivarius*, isolated from wild boar feces displayed a pH-dependent inhibitory activity against BCG and influenced its intake by porcine blood phagocytes in a species-dependent manner. All lactobacilli showed a very significant bactericidal effect against BCG at low pH, but only isolates of *L. plantarum* and *L. casei* displayed such antimycobacterial activity at neutral pH. The genomes of these isolates revealed the presence of two-peptide bacteriocins whose precursor genes up-regulate in the presence of BCG cells. Furthermore, *L. plantarum* reduced significantly the BCG phagocytic intake, whereas *L. casei* had the opposite effect. *L. salivarius* had no significant influence on the phagocytic response to BCG.

**Conclusions**: Our *in vitro* results show that lactobacilli isolated from wild boar antagonize BCG as a consequence of their antimycobacterial activity and a competitive phagocytic response. These findings suggest that commensal bacteria could play a beneficial role in influencing the outcome of bTB dissemination. Further work with lactobacilli as a potential competitive pressure to control bTB will need to take into account the complex nature of the commensal microbiome, the specific immunity of the wild boar and the *in vivo* infection context with pathogenic strains of *M. bovis*.

## Introduction

Bovine tuberculosis (bTB) is a chronic infectious disease caused by *Mycobacterium bovis* that affects livestock production, leading to significant economic losses worldwide (Ayele et al., 2004; Naranjo et al., 2008). Despite decades-long eradication campaigns bTB is still very prevalent in some European countries including Spain, where wildlife reservoirs of *Mycobacterium bovis* have been confirmed (Corner, 2006; Naranjo et al., 2008). Wild ungulates such as the European wild boar (*Sus scrofa*) have been reported to show a high bTB prevalence across Spain (Parra et al., 2006). In this respect, the scientific evidence suggests that the cases of bTB infection occur more frequently in regions with higher densities of wild boar (Aranaz et al., 2004). Therefore, wild boar seems to be an additional obstacle for complete bTB eradication in Spain.

Together with epidemiological and ecological studies, a better knowledge on the pathology and transmission of *M. bovis* infection is essential to determine the significant role of wildlife as a bTB reservoir. Like any other species of the *Mycobacterium tuberculosis* complex, *M. bovis* cells are initially phagocytized by macrophages, where they are able to survive, replicate and disseminate into different anatomical parts of the body (Cosma et al., 2004). When the macrophages transport the bacteria into deeper tissues, additional macrophages gather around the individual infected foci to form granulomas, which are organized immune complexes of differentiated macrophages, lymphocytes, and neutrophils. Neutrophils are also present at early stages of the infection. Despite the formation of granulomas in response to *M. bovis* infection, bacterial persistence and reinfection occur, but with no clear symptoms, which is the central paradox of bTB immunity (Cosma et al., 2003). Some studies have revealed that a significant number of animals within a *M. bovis*-infected cattle group display no clinical signs, but present generalized bTB lesions at post-mortem examination (Menin et al., 2013). In this respect, wild boars are likely to be no different. Very recently, generalized bTB has been reported in wild boar, including cases with thoracic and abdominal lesions, usually in the bronchial and mesenteric lymph nodes (Martin-Hernando et al., 2007; Matos et al., 2016).

The absence of clinical symptoms in wild boar with generalized bTB, which usually shed a large amount of *M. bovis* cells to the environment (Santos et al., 2015), and the fact that bTB-like lesions are present in more than one anatomical region, complicate diagnosis, the understanding of the infection route, and, subsequently, disease control. It is assumed that *M. bovis* enters through the oral mucosa and tonsils *via* food, water or air borne, and disseminate from the mandibular lymph nodes (Martin-Hernando et al., 2007; Menin et al., 2013; Matos et al., 2016). However, the variable distribution of lesions suggests that; first, it is not possible to elucidate whether the respiratory or the digestive route is more relevant for bTB infection in wild boar; and, second, the immune response of each individual may have an influence on the outcome of the disease. Although the reasons for this variability in lesion distribution have not been accounted for conclusively, one explanation could be due to the contribution of the wild boar genetic variability (Acevedo-Whitehouse et al., 2005). Another possibility that has not been explored before is the influence of the host-beneficial commensal-primed immune barrier.

Pathogenic mycobacteria have evolved to evade host defenses in order to reach and exploit its replicative niche. However, this evasion may be unsuccessful if host commensal bacteria exert an extra immune-competitive pressure (Cambier et al., 2014). The host-commensal alliance forms a barrier that pathogens such as *M. bovis* must circumvent to survive and replicate. Commensal bacteria populate abundantly the mucosal surfaces of pigs and are composed of different species that predominantly belong to two dominant bacterial phyla: firmicutes and bacteroidetes (Xiao et al., 2016). In particular, one of the most abundant beneficial groups within firmicutes is the genus *Lactobacillus* (Mann et al., 2014). Lactobacilli are normal inhabitants of the intestinal tract of humans and mammals but also of the tonsillar crypts, nasopharynx, and upper respiratory tract, among others (Martens et al., 2018). Several species of *Lactobacillus* modulate immune responses as they interact regularly with epithelial cells and antigen-presenting cells such as macrophages (Mohamadzadeh et al., 2005; Rocha-Ramirez et al., 2017). The specific mechanisms of such immune modulations are unknown but it has been demonstrated that components of the cell wall and membrane of lactobacilli, such as pili (fimbriae), peptidoglycans, lipoteichoic acids, and exopolysaccharides play an important role in activating phagocytic innate immune cells (Hevia et al., 2015).

The functional involvement of lactobacilli in the modulation of immune responses is critical to maintain homeostasis, particularly in the gut (Diep et al., 2009; Gueimonde et al., 2013; Liu et al., 2014). Furthermore, *Lactobacillus* spp. are also essential to support a beneficial microbial balance in the host as they prevent against colonization of opportunistic pathogens (Martin et al., 2013). It is very well documented that lactobacilli produce antimicrobial metabolites such as organic acids, hydrogen peroxide, ethanol, and bacteriocins to compete for nutrients (Pessione, 2012); and some of these compounds can be active against *M. bovis* (Stedman et al., 2018). In particular, bacteriocins produced by *Lactobacillus* spp. have been reported to display antimicrobial activity against *M. tuberculosis* (Todorov et al., 2008, 2014). Bacteriocins are antimicrobial peptides that can be classified into three different classes depending on their chemical structure and size: (1) class I, post-translationally modified peptides (e.g. nisin); (2) class II, unmodified peptides smaller than 10 kDa, including the pediocin-like (class IIa); the two peptide- (class IIb); the leaderless (class IIc); and the single peptide-(class IId) bacteriocins; and (3) class III, unmodified peptides larger than 10 kDa with bacteriolytic (bacteriolysins) or non-lytic mechanism of action (Alvarez-Sieiro et al., 2016). Furthermore, it has been reported that bacteriocins from lactobacilli contribute to immunomodulatory effects on peripheral blood mononuclear cells and dendritic cells (Hegarty et al., 2016).

The fact that *M. bovis* may encounter lactobacilli in the gut or respiratory tract of the host while exploiting macrophages as its optimal niche for survival and replication and the evidence that lactobacilli are able to interact with macrophages and display antimycobacterial activity, suggest that lactobacilli could act as a key beneficial microbe against bTB dissemination as a consequence of an antagonistic competition for nutrients and macrophages. The aim of this study was to determine whether *Lactobacillus* spp. isolated from wild boar are capable of displaying this antagonistic role against the surrogate *M. bovis* strain Bacillus Calmette-Guerin (BCG). We isolated three *Lactobacillus* species from wild boar feces (*L. plantarum*, *L. salivarius* and *L. casei*) that were co-cultured with BCG to evaluate their potential antagonistic influence on the survival rate of *M. bovis*. To study their potential as macrophage competitors, porcine blood phagocytes were exposed to BCG in the presence of the three *Lactobacillus* species to quantify the BCG intake. Our *in vitro* results demonstrate that lactobacilli isolated from wild boar have the capacity to influence the phagocytic response to BCG and its survival in a species-dependent manner.

## Materials and Methods

### Isolation of Lactobacilli From Wild Boar Fecal Samples

Fecal samples were collected from a total of 30 wild boar from three fenced game estates located in mid-western Spain. These three states are surrounded by areas with a significant clinical history of bTB. In all cases, the fecal samples were taken using rectal swabs with AMIES transport medium (Deltalab) and stored at 4°C for a maximum of 1–2 days until further processing. The collected rectal swabs were immersed in sterile peptone water (Oxoid) to prepare serial dilutions that were spread onto De Man, Rogosa, and Sharpe (MRS) agar plates for the isolation of *Lactobacillus* spp. Plates were incubated under microaerophilic conditions at 37°C for 48–60 h and 50 colonies showing different morphologies (where possible) were then selected of each sample from plates containing 20–100 colonies. Individual colonies were inoculated to Thermo Scientific™ Nunc™ MicroWell™ 96-well plates with MRS broth at 37°C for 48 h under microaerophilic conditions.

### Antimycobacterial Screening and Identification of Lactobacilli Isolates

Isolates propagated in MRS broth were tested against *Mycobacterium smegmatis* mc^2^155, a non-pathogenic fast-growing mycobacteria species that facilitates rapid antimycobacterial screening (Stedman et al., 2018). The MRS cultures present in the 96-well plates were replica plated with a Scienceware^®^ 96-well replicator (Sigma-Aldrich) onto Tryptone Soya Agar (TSA, Oxoid) plates that had previously been swabbed with a culture of *M. smegmatis* mc^2^155 (Supplementary Figure S1). *M. smegmatis* was cultured in Tryptone Soya Broth (TSB, Oxoid) supplemented with 0.05% of Tween 80 (Sigma) in an orbital shaker at 37°C for 48 h. The TSA plates were then incubated at 37°C for 48 h and the cultures displaying antimycobacterial activity selected for colony isolation on MRS agar plates following an incubation of 48 h at 37°C. Colonies were identified using gram-staining (positive rods), oxidase/catalase tests (negative) and 16S rRNA sequencing (LGC Genomics) before a final selection of the most representative lactobacilli isolates comprising different strains and species from different animals belonging to different estates.

### Growth Conditions for Lactobacilli Isolates and *Mycobacterium Bovis* Bacillus Calmette-Guerin Strains

Colonies from the selected lactobacilli isolates were grown in MRS broth/agar (Oxoid) at 37°C without any aeration for 24–48 h. Two *Mycobacterium bovis* Bacillus Calmette-Guérin (BCG) strains were used to determine the antagonistic effect of the lactobacilli isolates against *M. bovis*: BCG Pasteur and BCG Δ*leuD* pAS^OriM^XF (Brosch et al., 2000; Stedman, 2017). Both BCG strains were cultured in Middlebrook 7H9 (Difco) broth supplemented with 10% Oleic acid-Albumin-Dextrose-Catalase enrichment (OADC, Sigma-Aldrich), 0.05% Tween 80 (Sigma-Aldrich), 0.2% glycerol and 40 μg/ml kanamycin at 37°C in an orbital shaker at 225 rpm for 5–7 days. BCG Δ*leuD* pAS^OriM^XF was generated by transforming the BCG Pasteur Δ*leuD* strain with pAS^OriM^XF as previously described (Stedman, 2017). pAS^OriM^XF is a mycobacterial episomal vector that complements the leucine mutation to correct auxotrophy, and enable stable expression of GFP under the control of the constitutively expressed promoter pL5X (Borsuk et al., 2007). All bacterial strains were maintained as −80°C frozen stocks in their appropriate media with the addition of 15% glycerol.

### Co-cultures of Lactobacilli and Bacillus Calmette-Guerin

Cell pellets of the selected lactobacilli and the BCG strains were obtained from cultures at their early stationary phase of growth following centrifugation at 8,000 rpm for 5 min. The BCG pellets were resuspended with each *Lactobacillus* pellet using Mueller-Hinton (MH) broth supplemented with 10% Oleic acid-Albumin-Dextrose-Catalase (OADC) enrichment, 0.1% Tween 80 (Tw80) and 0.2% glycerol (Gly) (MH-OADC-Tw80-Gly), to generate co-cultures at an initial concentration of 5 × 10^8^ CFU/ml for BCG and 5 × 10^6^ CFU/ml for the lactobacilli. In a previous study we reported that MH supplemented with OADC, tween, and glycerol is the optimal broth to support the growth of both lactobacilli and BCG when grown as mono-cultures for 48 h (Stedman et al., 2018). The co-cultures were then grown in an orbital shaker at 37°C for 48 h and samples were collected at 0, 24, and 48 h post-incubation to determine BCG survival rate.

### Bacillus Calmette-Guerin Survival Rate in Co-cultures

The survival rate of BCG in co-cultures with the lactobacilli isolates was determined simultaneously by total bacterial counts and GFP expression. Total bacterial counts were calculated from co-cultures of the two selected BCG strains using a volume of 100 μl on mycobacteria-selective agar plates (Middlebrook 7H10 supplemented with 5% OADC and 0.2% glycerol). Bacterial counts were also carried out for lactobacilli on their corresponding selective MRS agar plates and presented as log_10_ CFU/ml. Colony forming units (CFU)/ml were calculated by dividing the number of colonies present on a plate by the dilution factor of the sample and the volume transferred to the selected plate. The GFP expression was monitored in co-cultures with the auxotrophy correction leucine auxotrophic strain BCG Δ*leuD* pAS^OriM^XF, which maintains the GFP plasmid in broth without using any antibiotics (Stedman et al., 2018). Aliquots of 100 μl were transferred into Thermo Scientific Nunc MicroWell™ 96-well plates to measure fluorescence emission at 485/535 nm in a DTX 880 Multimode Detector microplate reader (Beckman Coulter). GFP expression was then indicated as fluorescence units (FU).

### pH in Co-cultures

The pH of all co-cultures was measured using a Hanna pH meter at time points 0 and 48 h, starting from an initial pH of 7 for the media, to determine the effect of pH on the survival rate of BCG while co-culturing with lactobacilli samples. Furthermore, the survival rate of BCG was monitored as a monoculture at pH7 and pH4.5 in MH-OADC-Tw80-Gly to evaluate the influence of acidic pH on the growth of BCG. The monitoring was carried out based on bacterial counts (log_10_ CFU/ml) and GFP expression (FU) from the two BCG strains as explained above.

### Accumulation of Anti-mycobacterial Metabolites From Lactobacilli in Mono-Cultures and Co-cultures With Bacillus Calmette-Guerin

In order to determine the effect of antimicrobial compounds produced by the lactobacilli isolates on BCG growth, cell pellets of BCG Δ*leuD* pAS^OriM^XF were resuspended in cell-free supernatants collected from 24 h cultures of lactobacilli in MH-OADC-Tw80-Gly at a concentration of 5 × 10^8^ CFU/ml. BCG pellets were also resuspended in cell-free supernatants obtained from 24 h co-cultures of lactobacilli and BCG at the same final concentration using the same supplemented MH broth to evaluate the influence of BCG on the production of antimycobacterial compounds by the lactobacilli isolates. For both sets of experiments cell pellets were obtained after centrifugation at 8,000 rpm for 5 min, and the pH of the supernatants was adjusted to 7 and 4.5 to consider any possible synergistic antimicrobial effects at low pH. The controls consisted of BCG cultures in cell-free supernatants obtained from 24 h-incubated MH-OADC-Tw80-Gly broth or 24 h-BCG monocultures in MH-OADC-Tw80-Gly. The survival rate of BCG was then determined as GFP expression (FU) as indicated above.

### Nutrient Alteration Caused by Lactobacilli in Mono-Cultures and Co-cultures With Bacillus Calmette-Guerin

Additional GFP experiments with the collected supernatants indicated above were carried out at pH7 but mixed with MH-OADC-Tw80-Gly at a ratio of 1:1 to evaluate the influence of nutrient alteration on the survival rate of BCG. In this case, the supernatants for the controls were also adjusted to pH7 and supplemented with MH-OADC-Tw80-Gly at 1:1.

### Influence of Bacillus Calmette-Guerin Metabolites on the Production of Anti-mycobacterial Metabolites by Lactobacilli

In order to test whether the antimycobacterial activity observed from co-cultures of BCG with our lactobacilli isolates could be due to an inducing effect from BCG metabolites we monitored the survival rate of BCG-GFP strain in supernatants derived from lactobacilli cultures in supernatants from BCG cultures. Briefly, BCG-GFP cells were resuspended in cell-free supernatants collected from cultures of lactobacilli after 24 h of incubation in supernatants from a 5 day-old BCG culture. Our controls consisted of BCG-GFP cells resuspended in cell-free supernatants obtained from 24 h cultures of lactobacilli in BCG supernatants supplemented with MH-OADC-Tw80-Gly at a ratio of 1:1, and also in MH-OADC-Tw80-Gly. For the three sets of experiments the pH of the supernatants was adjusted to 7. The survival rate of BCG was then determined as GFP expression (FU) as indicated above.

### Genome Sequencing, Assembly, and Annotation of the Lactobacilli Isolates

The lactobacilli isolates were sent as pure isolated colonies on MRS agar plates to MicrobesNG, University of Birmingham, UK, where genome sequencing was carried out using the Illumina MiSeq platform. The DNA was extracted using the EZNA^®^ Bacterial DNA kit (Omega Bio-Tek, USA) and the library preparation was carried out with the Nextera™ XT Library Prep Kit. The DNA from each isolate was sequenced using 2 × 250 bp paired-end reads and put through a standard analysis pipeline. The quality of the generated reads, which were first trimmed using Trimmomatic, was assessed using in-house scripts. Genomes were assembled from the paired-end reads using Shovill version 1.0.4^1^ with SPAdes 3.13.0 as assembly module (Bankevich et al., 2012), using default settings. Assembly quality was assessed by N50 and L50, using Quast version 4.5 (Gurevich et al., 2013), and the genome assemblies were annotated using Prokka version 1.13 (Seemann, 2014). Sequencing reads, genome assemblies and metadata have been uploaded onto Genbank in BioProject PRJNA544176.

### Identification of Genes and Clusters Associated With the Synthesis of Antimicrobial Metabolites and Macrophage Activation in Lactobacilli

The genome assemblies were uploaded on the online BAGEL3 software (van Heel et al., 2013) to identify gene clusters involved in the biosynthesis of bacteriocins. This software allows for a rapid and reliable identification of all classes of bacteriocin clusters, which are usually composed of genes encoding for the bacteriocin precursor (pre-bacteriocin), proteins involved in the transport and processing of the pre-bacteriocin as an active bacteriocin, posttranslational modification enzymes and immunity proteins. Furthermore, the genome annotations were used to confirm the presence or absence of fructose-6-phosphate aldolase and phosphoketolase, 2 enzymes that are involved in the 2 main glycolytic pathways in LAB: the Embden-Meyerhof pathway (EMP) and the phosphoketolase pathway (PKP), respectively (Papagianni, 2012a). In general, homofermentative LAB convert carbohydrates into lactate using EMP, whereas heterofermentative LAB produce lactate, acetate, ethanol, and carbon dioxide as antimicrobial metabolites *via* PKP. The genome annotations were also used to identify genes associated with hydrogen peroxide (H_2_O_2_) production, including genes that encode for pyruvate oxidase (Pox), lactate oxidase (Lox), and NADH oxidases. Genes encoding for NADH peroxidases were also included in the search for H_2_O_2_ production markers as H_2_O_2_ may accumulate is species that lack these hydrogen peroxide-scavenging enzyme (Hertzberger et al., 2014). We finally carried out a search on the genome annotations to detect cell wall and membrane compounds associated with macrophage activation *via* TLR and/or phagocytic receptors, including exopolysaccharides (EPS), fimbrial precursors, lipoteichoic acid (LTA), wall teichoic acid (WTA), and adhesins, as previously described for lactobacilli (Van Tassell and Miller, 2011; Sengupta et al., 2013; Hevia et al., 2015).

### Level of Expression of Genes Encoding for Hypothetical Bacteriocin Precursors Identified in the Lactobacilli Genomes

The level of expression of transcripts from bacteriocin precursor genes was determined by Reverse Transcription-PCR (RT-PCR) analysis. Total RNA was extracted from cultures of lactobacilli in the absence or presence of BCG cells at two different concentrations (10^6^ and 10^7^ CFU/ml) using the High Pure RNA Isolation Kit (Roche Diagnostics Limited) as recommended by the manufacturer’s instructions. All culture samples were collected during exponential growth at an OD_600nm_ of 0.6. RNA was treated with TURBO DNase (Ambion) to eliminate traces of contaminating genomic DNA and the DNA-free RNA was reverse transcribed using the SuperScript^®^ III First-Strand Synthesis System from Invitrogen. Reverse transcription (RT)-PCR analyses were performed on the QuantStudio 7 Flex Real-Time PCR system (Applied Biosystems) using the SYBR Master Mix (Applied Biosystems) and the specific primers for the selected transcripts (Table 1). Transcripts were amplified on a 96-well format plate by using three technical replicates of samples obtained from at least two biological replicates. We then used the ΔΔCT method to calculate the relative quantification of mRNA expression from cultures exposed to BCG cells by comparison with those with no BCG added. The levels of gene expression were normalized using the housekeeping transcripts *gyrA* and *dnaG* for each of the selected species as previously recommended (Rocha et al., 2015) and indicated as log_2_.

**Table 1:** Primers used for the amplification of transcritps derived from the class II bacteriocin precursor genes of *L. plantarum*, *L. casei,* and *L. salivarius*.

Gene	Bacteriocin	Primers	
*plnE*	Plantaricin E, *L. plantarum*	Fw: caatattccaggttgccgca	Rv: gaatgcctgcaactgaacca
*plnF*	Plantaricin F, *L. plantarum*	Fw: atttcaggtggcgttttcca	Rv: aatcctcggacagcgctaat
*plnJ*	Plantaricin J, *L. plantarum*	Fw: gccagcttcgccatcataaa	Rv: aggatttggatgtagtagatgca
*plnK*	Plantaricin K, *L. plantarum*	Fw: ttgaaccaccaagcacgg	Rv: ttgaagaattaactgctgacgc
*A*	Class IIb bacteriocin, *L. casei*	Fw: agttgtcaggggtttcaggt	Rv: ccgccgattatcccaaaagg
*B*	Class IIb bacteriocin, *L. casei*	Fw: gagccaagcgacgcaataaa	Rv: cgcctgcaacagttgtaaatg
*Tα*	Class IId bacteriocin, *L. salivarius*	Fw: gcaatcagaggaggaatggc	Rv: ccgatacaagccaatccacc
*Tβ*	Class IId bacteriocin, *L. salivarius*	Fw: gggaatggcattaattgggga	Rv: ggattaccgaaagctgcacc
*gyrA*	DNA topoisomerase, *L. plantarum*	Fw: tttaagtcgcaacaccgtgg	Rv: gattcctttggccgtacgac
*dnaG*	DNA primase, *L. plantarum*	Fw: agttggtagtcggtctggtg	Rv: cgcacctaaggatcagcaac
*gyrA*	DNA topoisomerase, *L. paracasei*	Fw: cttccacgcatgatgtcctg	Rv: cgccttcatgcacgttgata
*dnaG*	DNA primase, *L. paracasei*	Fw: cagttcggccaattgatcgt	Rv: cgactcgatccaggaatcca
*gyrA*	DNA topoisomerase, *L. salivarius*	Fw: gttttgccagcacgttttcc	Rv: tcccattacaattgcgccag
*dnaG*	DNA primase, *L. salivarius*	Fw: gcacaaagattcaacgtcgc	Rv: cgttctgctttctctgcctt

*gyrA and dnaG were selected as housekeeping transcripts as previously recommended (Rocha et al., 2015)*.

### Phagocytosis Assay With Porcine Blood Cells

Whole fresh blood was collected in heparinized tubes from healthy pigs at the Pirbright Institute (UK), where all animal procedures are covered by a license issued by the UK Home Office under the Animal (Scientific Procedures) Act1986. About 100 μl of whole blood, which is equivalent to approximately 10^6^ leukocytes, were mixed 1:1 with 10 mM EDTA (EDTA-PBS) and challenged with BCG in combination with lactobacilli at a multiplicity of infection (MOI) of 10 bacteria per 1 blood cell. The bacterial combinations were prepared in PBS and contained the GFP-expressing BCG strain Δ*leuD* pAS^OriM^XF and each of the three *Lactobacillus* species. The same MOI was used for the preparation of control samples, which were obtained using monocultures of BCG Δ*leuD* pAS^OriM^XF, BCG Pasteur and lactobacilli. The blood cells and bacteria were incubated in an orbital shaker at 37°C for 30 min and subsequently lysed with 1x RBC lysis solution (Biolegend) following incubation at room temperature for 15 min. The cells were then washed twice with EDTA-PBS, resuspended in PBS and acquired on the flow cytometer BD FACS Celesta. FACS sorting based on forward (FSC) and side (SSC) scatter was used to distinguish the main blood cell populations based on their size and granularity (lymphocytes vs. phagocytes), while the FITC channel let us measure the GFP levels in blood cells that bind (e.g. lymphocytes) and/or phagocytize (e.g. phagocytes including monocytes and PMNs such as neutrophils). The resulting SCC/GFP plot was then used to evaluate the influence of lactobacilli on the BCG intake by phagocytes.

### Statistical Analysis

Statistical analysis was performed using GraphPad Prism version 7.00 for Windows (GraphPad Software, La Jolla California USA, www.graphpad.com). Data are mean ± SD, representing three biological replicates, unless indicated. Differences between time-points for the same samples were analyzed by the Student *t*-test.

## Results

### Lactic Acid Bacteria Isolated From Wild Boar Show Anti-mycobacterial Activity and Are Identified as Lactobacilli

A total of 16 strains isolated from wild boar feces on MRS selective agar displayed antimicrobial activity against *M. smegmatis* mc^2^155 as illustrated in Supplementary Figure S1. These 16 isolates were identified as *L. plantarum*, *L. salivarius, L. paracasei*, of which 6 were selected for further experiments as they represented 3 different species, with two strains of the same species (where possible) from at least two different animals and 3 different game estates. The six selected lactobacilli were *L. plantarum* C1, *L. plantarum* EML1, *L. plantarum* SA3, *L. salivarius* C2, *L. salivarius* C12, and *L. paracasei* SA5. Further species identification was carried out using StrainSeeker and ANItools (Han et al., 2016; Roosaare et al., 2017), two internet tools that allow for fast species identification by genome comparison with closely-related bacterial strains. With the exception of isolate SA5, the species of all isolates were confirmed. The genomes of our *L. plantarum* isolates form a phylogenetic clade with those of *L. plantarum* WCFS1 and B21 (isolates C1 and EML1) (Kleerebezem et al., 2003; Golneshin et al., 2015) and *L. plantarum* P-8 (isolate SA3) (Golneshin et al., 2015). We also observed two more clades between our isolates C2 and C12 and the *L. salivarius* strains JCM1046 and CECT5713 (Jimenez et al., 2010; Raftis et al., 2014), respectively. With regards to isolate SA5 this was finally determined to be *L. casei* instead of *L. paracasei* as its genome shares a phylogenetic clade with the genome of *L. casei* BL23 (Maze et al., 2010).

### Lactobacilli Reduce Survival Rate of *M. bovis* Bacillus Calmette-Guerin and Lower pH in Co-cultures

**Figure 1:**
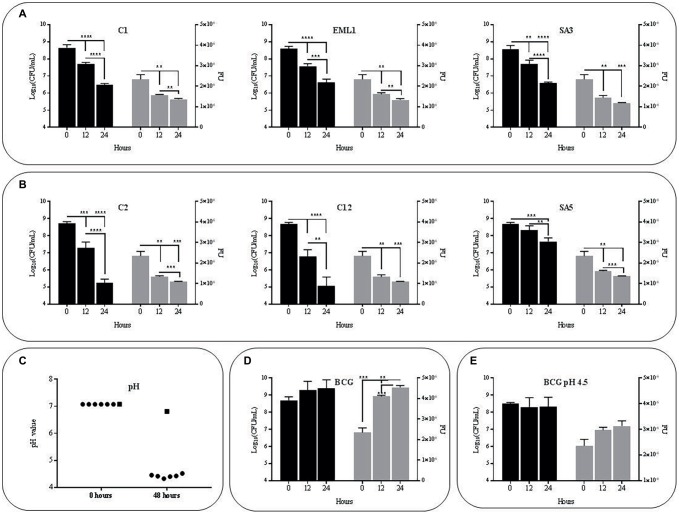
Survival rate and acidity of BCG cultures after 48 h (h) of incubation in MH broth with OADC (10%), Tween 80 (0.1%) and glycerol (0.2%).The survival rate was monitored as bacterial counts (log_10_CFU/ml indicated with black bars) and GFP expression (FU indicated with gray bars), while acidity was measured as pH values. Data are mean ± SD with statistical analysis (Student’s *t*-test, **p < 0.01, ***p < 0.005, ****p < 0.001). **(A)** Survival rate of BCG in co-cultures with the *L. plantarum* strains C1, EML1 and SA3 after 0, 24 and 48 h of incubation. **(B)** Survival rate of BCG in co-cultures with the *L. salivarius* strains C2, C12 and *L. casei* SA5 after 0, 24 and 48 h of incubation. **(C)** Acidity of BCG co-cultures with lactobacilli strains C1, EML1, SA3, C2, C12 and SA5 (indicated as circles from left to right) and BCG monocultures (indicated as a square) after 0 and 48 h of incubation. **(D)** Survival rate of BCG mono-cultures after 0, 24 and 48 h of incubation from an initial pH of 7. **(E)** Survival rate of BCG mono-cultures after 0, 24 and 48 h of incubation from an initial pH of 4.5.

The selected isolates *L. plantarum* C1/EML1/SA3, *L. salivarius* C2/C12 and *L. casei* SA5 were cultured simultaneously with BCG Pasteur and the GFP-BCG strain Δ*leuD* pAS^OriM^XF (Brosch et al., 2000; Stedman, 2017) to determine the influence of lactobacilli on the survival rate of *M. bovis*. We monitored the survival rate of BCG by measuring total BCG Pasteur counts and GFP emission from BCG Δ*leuD* pAS^OriM^XF for 48 h, as illustrated in Figure 1. All the lactobacilli reduced BCG counts and GFP emission significantly after 24 and 48 h (Figures 1A,B). The pH of the co-cultures was also recorded in all the co-cultures after 48 h. We observed a pH decrease below 4.5, with no significant differences between co-cultures (Figure 1C). As pH of BCG monocultures slightly decreased from 7 to 6.8 we next tested whether acidic pH could account for the reduction observed in BCG viability. However, both total bacterial counts and GFP emission of BCG as a monoculture showed no detrimental changes at pH7 or pH4.5 after 24 and 48 h (Figures 1D,E). These results confirm that low pH may be insufficient on its own to cause a negative effect on BCG survival.

### Metabolites From Lactobacilli Reduce Survival Rate of Bacillus Calmette-Guerin at Low pH

**Figure 2:**
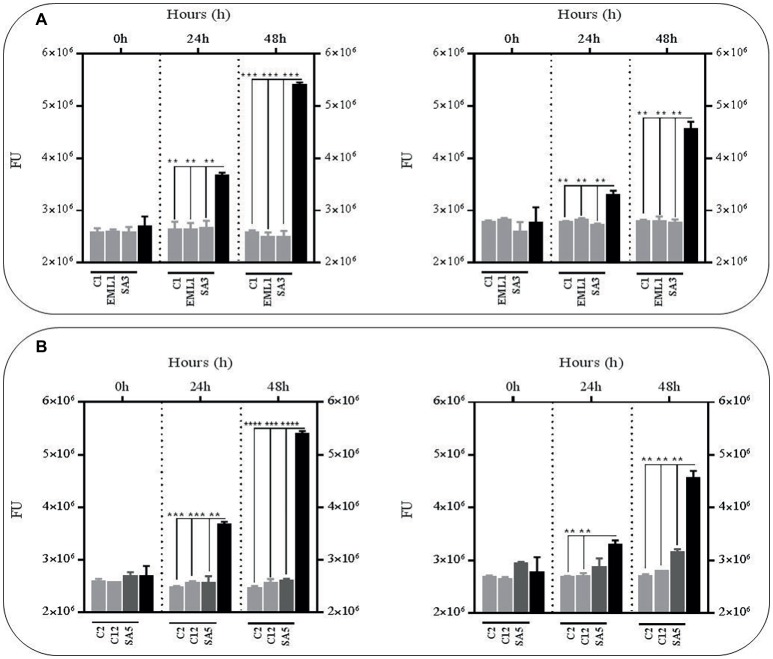
Survival rate of BCG after 48 h (h) of incubation in acidic cell-free supernatants (pH 4.5) that were obtained from 24 h mono-cultures of lactobacilli (left hand side) or 24 h co-cultures of BCG with lactobacilli (right hand side) in MH broth with OADC (10%), Tween 80 (0.1%) and glycerol (0.2%). The BCG survival rate for both conditions was monitored as GFP expression (FU indicated with gray bars) and compared to their corresponding controls represented with black bars. The controls were BCG grown in cell-free supernatants obtained from a 24 h-incubated MH broth with OADC (10%), Tween 80 (0.1%) and glycerol (0.2%) (left hand side) and 24 h BCG monocultures in MH broth with OADC (10%), Tween 80 (0.1%) and glycerol (0.2%) (right hand side), both at pH 4.5. Data are mean ± SD with statistical analysis (Student’s *t*-test, **p < 0.01, ***p < 0.005, ****p < 0.001). **(A)** Survival rate of BCG after 0, 24 and 48 h of incubation in supernatants from mono-cultures of *L. plantarum* C1, EML1, SA3 (left hand side) or co-cultures of BCG with the *L. plantarum* strains (right hand side). **(B)** Survival rate of BCG after 0, 24 and 48 h of incubation in supernatants from mono-cultures of *L. salivarius* C2 and C12 and *L. casei* SA5 (left hand side) or co-cultures of BCG with the strains of *L. salivarius* and *L. casei* (right hand side).

In order to test whether the antimicrobial effects observed against BCG in the presence of lactobacilli was due to an accumulation of toxic metabolites, we monitored the survival rate of the GFP-BCG strain in cell-free supernatants obtained from mono-cultures of lactobacilli and co-cultures of lactobacilli and BCG after 24 h of incubation (Figure 2). For these experiments the survival rate was recorded as GFP emission as the anti-mycobacterial effect observed in the co-cultures resulted in a very significant positive correlation between total bacterial counts and GFP emission (Supplementary Figure S2). Cell-free supernatants were collected from both mono-cultures of lactobacilli and co-cultures of lactobacilli and BCG to determine whether the presence of BCG may act as an inducer on the production of antimicrobial compounds by lactobacilli. We established 24 h as the collection time point for the supernatants since lactobacilli experience a significant log increase in bacterial counts over the first 24 h (Supplementary Figure S3). The bacterial counts recorded for all lactobacilli cultures, either as mono-cultures or co-cultures, were no significantly different. No significant differences were observed in the pH recorded for both lactobacilli culture conditions after 24 h either (data not shown). As illustrated in Figure 2, very significant reductions were observed with all the culture supernatants, either from mono-cultures or co-cultures with BCG, especially after 48 h. This demonstrated that the anti-mycobacterial activity of lactobacilli in co-cultures could be due to the combined effect of acidic pH and accumulation of antimicrobial metabolites derived from lactobacilli.

### Metabolites From *L. plantarum* Co-cultures With Bacillus Calmette-Guerin Reduce the Survival Rate of Bacillus Calmette-Guerin Regardless of pH and Nutrient Supplementation

**Figure 3:**
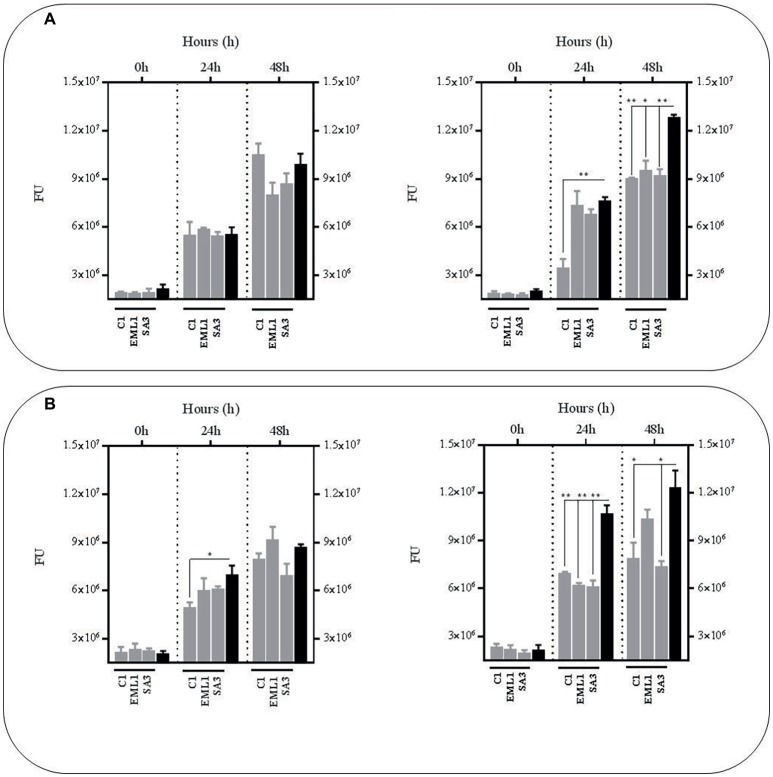
Survival rate of BCG after 48 h (h) of incubation in neutralized cell-free supernatants (pH 7) that were obtained from 24 h mono-cultures of *L. plantarum* C1, EML1 and SA3 (left hand side) or 24 h co-cultures of BCG with the *L. plantarum* strains (right hand side) in MH broth with OADC (10%), Tween 80 (0.1%) and glycerol (0.2%). The BCG survival rate for both conditions was monitored as GFP expression (FU as gray bars) and compared to their corresponding controls represented with black bars, with or without nutrient supplementation. The controls were BCG grown in cell-free supernatants obtained from a 24 h-incubated MH broth with OADC (10%), Tween 80 (0.1%) and glycerol (0.2%) (left hand side) and 24 h BCG monocultures in MH broth with OADC (10%), Tween 80 (0.1%) and glycerol (0.2%) (right hand side), both at pH 7. Data are mean ± SD with statistical analysis (Student’s *t*-test, *p < 0.05, **p < 0.01). **(A)** Survival rate of BCG after 0, 24 and 48 h of incubation in supernatants from *L. plantarum* mono-cultures (left hand side) or co-cultures of BCG with the *L. plantarum* strains (right hand side). **(B)** Survival rate of BCG after 0, 24 and 48 h of incubation in supernatants from *L. plantarum* mono-cultures (left hand side) or co-cultures of BCG with the *L. plantarum* strains (right hand side) that were supplemented with MH broth with OADC (10%), Tween 80 (0.1%) and glycerol (0.2%) at a ratio of 1:1. The supernatants for the controls were also supplemented with MH broth with OADC (10%), Tween 80 (0.1%) and glycerol (0.2%) at 1:1.

In order to confirm the anti-mycobacterial effect of metabolites produced by lactobacilli, we carried out the same experiments as described above with the supernatants, either from mono-cultures or co-cultures with BCG, but at pH 7 to exclude the positive antimicrobial effect of acidic pH. We also included a third experimental condition by supplementing the supernatants with fresh co-culture media to determine the influence of nutrient alteration caused by lactobacilli metabolism. Due to logistic reasons we started with the *L. plantarum* strains. As illustrated in Figure 3, no significant reductions were observed in BCG survival using the supernatants obtained from the *L. plantarum* mono-cultures, either with or without nutrient supplementation. However, the reduction was very significant when we used the supernatants from the co-cultures of the three *L. plantarum* strains with BCG, especially with supplementation after 24 h, but also after 48 h without any supplementation. These results suggest that our *L. plantarum* strains over-produce certain anti-mycobacterial metabolites in the presence of BCG. Furthermore, this induced antimicrobial activity seems to be stable at different ranges of pH and nutrient composition.

### The Anti-mycobacterial Effect of Metabolites From *L. salivarius* and *L. casei* Depends on pH but Also on the Presence of Bacillus Calmette-Guerin if Derived From *L. casei*

**Figure 4:**
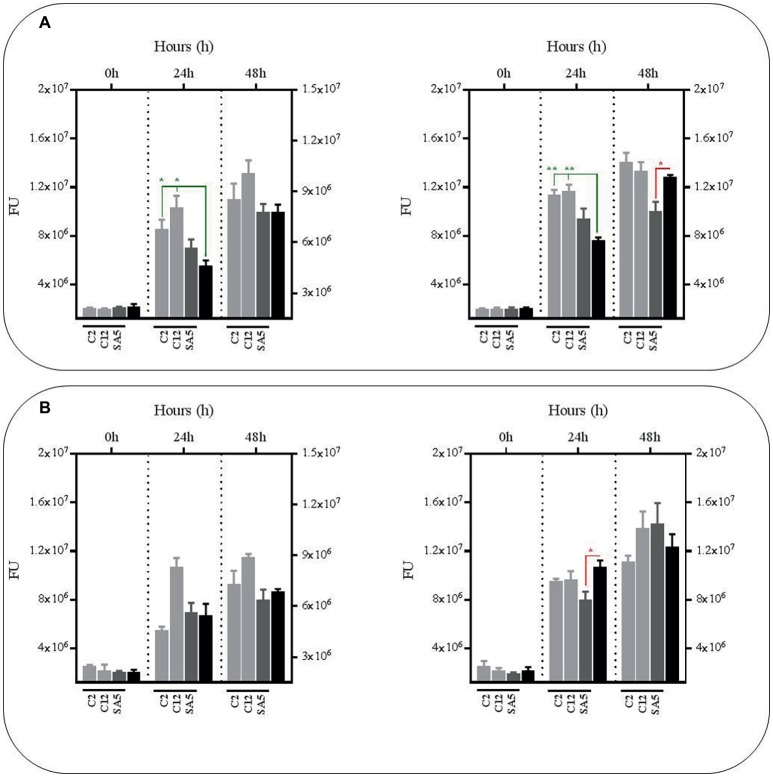
Survival rate of BCG after 48 h (h) of incubation in neutralized cell-free supernatants obtained from 24 h mono-cultures of *L. salivarius* C2 and C12 and *L. casei* SA5 (left hand side) or 24 h co-cultures of BCG with the strains of *L. salivarius* and *L. casei* (right hand side) in MH broth with OADC (10%), Tween 80 (0.1%) and glycerol (0.2%). The BCG survival rate for both conditions was monitored as GFP expression (FU as light gray bars for *L. salivarius* and dark gray bars for *L. casei*) and compared to their corresponding controls represented with black bars, with or without supplementation. The controls were BCG grown in cell-free supernatants obtained from a 24 h-incubated MH broth with OADC (10%), Tween 80 (0.1%) and glycerol (0.2%) (left hand side) and 24 h BCG monocultures in MH broth with OADC (10%), Tween 80 (0.1%) and glycerol (0.2%), both at pH 7 (right hand side). Data are mean ± SD with statistical analysis (Student’s *t*-test, *p < 0.05, **p < 0.01) and significant GFP differences are indicated in green (increase) or red (decrease). **(A)** Survival rate of BCG after 0, 24 and 48 h of incubation in supernatants from mono-cultures of *L. salivarius* and *L. casei* (left hand side) or co-cultures of BCG with the strains of *L. salivarius* and *L. casei* (right hand side). **(B)** Survival rate of BCG after 0, 24 and 48 h of incubation in supernatants from mono-cultures of *L. salivarius* and *L. casei* (left hand side) or co-cultures of BCG with the strains of *L. salivarius* and *L. casei* (right hand side) that were supplemented with MH broth with OADC (10%), Tween 80 (0.1%) and glycerol (0.2%) at a ratio of 1:1. The supernatants for the controls were also supplemented with MH broth with OADC (10%), Tween 80 (0.1%) and glycerol (0.2%) at 1:1.

As observed with the *L. plantarum* supernatants no significant reductions were observed in BCG survival using the supernatants obtained from mono-cultures of *L. salivarius* and *L. casei* at pH7, either with or without nutrient supplementation (Figure 4). In fact, the BCG survival increased in the supernatants from the *L. salivarius* mono-cultures without supplementation. The same observation was recorded with the supernatants collected from the co-cultures of *L. salivarius* and BCG, confirming a pH-dependency on the anti-mycobacterial effect from the *L. salivarius* strains. On the contrary, the survival rate of BCG decreased slightly in the supernatants derived from co-cultures of *L. casei* and BCG, suggesting a role of BCG as an inducer on the production of antimicrobial compounds in *L. casei*. Nevertheless this induction seems to be significantly lower than that observed with *L. plantarum*.

### Metabolites From Bacillus Calmette-Guerin Have No Influence on the Production of Anti-mycobacterial Metabolites by *L. plantarum* and *L. casei*

In order to test whether the induced antimycobacterial activity observed from co-cultures of BCG with the *L. plantarum* strains and *L. casei* SA5 was dependent on the presence of BCG metabolites rather than cells, we monitored the survival rate of BCG-GFP strain in cell-free supernatants derived from cultures of *L. plantarum* and *L. casei* that were propagated for 24 h in cell-free supernatants from 5 day-old BCG cultures. We also included two additional experimental conditions by supplementing the supernatants with fresh co-culture media but also by growing the lactobacilli in complete fresh co-culture broth to consider the possible negative influence of nutrient alteration in the growth of lactobacilli. The bacterial counts recorded for the 3 different types of lactobacilli cultures were not significantly different (Supplementary Figure S4). The pH of the supernatants from lactobacilli cultures propagated in BCG supernatants was slightly above 4.5 but no significant differences were observed when compared to the other lactobacilli cultures (data not shown). As illustrated in Supplementary Figure S5, no significant reductions were observed in BCG survival using the supernatants obtained from the *L. plantarum* cultures grown in supernatants from BCG cultures, either with or without nutrient supplementation. These data suggest that BCG metabolites have no inducing effect on the anti-mycobacterial activity from *L. plantarum* and *L. casei*. Subsequently, the higher antimicrobial effect observed in co-cultures of BCG with *L. plantarum* and *L. casei* when compared to mono-cultures could be due to the presence of BCG cells.

### Lactobacilli Harbor Genes Associated With the Synthesis of Antimicrobial Metabolites

The genome annotations let us identify the genes encoding for fructose-6-phosphate aldolase and phosphoketolase in all the lactobacilli isolates, demonstrating their role as facultative heterofermenters. Therefore, our isolates are able to convert carbohydrates into lactate using the EMP pathway and/or produce lactate in combination with ethanol, acetate and carbon dioxide as antimicrobial metabolites *via* the PKP pathway. Furthermore, all the isolates harbor genes associated with H_2_O_2_ production, e.g. including Pox, Lox, and/or NADH oxidases as well as genes encoding for the H_2_O_2_-scavenging enzyme NADH peroxidase. Interestingly, the BAGEL3 genome analysis identified gene clusters involved in the hypothetical synthesis of class II and class III bacteriocins, including single-peptide bacteriocins in *L. salivarius* C2 (Table 2); two-peptide bacteriocins in all *L. plantarum* strains and *L. casei* SA5 (Table 3), and bacteriolysins in all lactobacilli isolates (Table 4). The two single-peptide bacteriocin precursors identified in the genome of *L. salivarius* C2 (Tα, Tβ) show a very high homology with two class II bacteriocins of *L. salivarius* BGH01 (Busarcevic and Dalgalarrondo, 2012), while the two-peptide bacteriocin precursors of the *L. plantarum* genomes (*plnE*, *plnF*) are identical to the plantaricin precursor genes *plnE* and *plnF* of *L. plantarum* C11 (Anderssen et al., 1998). The two-peptide bacteriocin precursors found in the genome of *L. casei* SA5 (*A*, *B*) were also identical to two hypothetical class II bacteriocins of *Lactobacillus casei* DPC6800 (Stefanovic et al., 2016) and *Lactobacillus casei* UCD174 (Broadbent et al., 2012). On the other hand, the bacteriolysin genes that we identified in all the genomes encode for enzymes that hydrolyse cell wall peptidoglycans between N-acetylmuramic acid and N-acetyl-D-glucosamide, or N-acetylmuramoyl and L-aa residues. These enzymes normally have target recognition site and a catalytic domain that shows homology to endopeptidases, muramidases, or amidases (Cotter et al., 2005). Furthermore, unlike the class II bacteriocin clusters, the bacteriolysins have no specific immunity genes that accompany the bacteriocin precursor genes as they rely on modifications of the producer cell wall to impart resistance. The identified bacteriolysins were very abundant in *L. salivarius* C2, followed by *L. salivarius* C12 and *L. casei* SA5. The *L. plantarum* strains only share two of them with the remainder of the isolates.

**Table 2:** Proteins encoded by the hypothetical single peptide-bacteriocin cluster of *Lactobacillus salivarius* C2.

ORF (gene)	Size (aa)	Function	Homologue	%	Reference
1 (*T2*)	87	Putative protein	*Lactobacillus salivarius* CCUG47825	96	(Harris et al., 2017)
2 (*T3*)	57	Bacteriocin-like peptide	*Lactobacillus salivarius* UCC118	96	(Flynn et al., 2002)
3 (*T4*)	85	Bacteriocin-like peptide	*Lactobacillus salivarius* ATCC11741	96	(Harris et al., 2017)
4 (*Ta*)	80	Subunit A bacteriocin ThmA	*Blp1a, Lactobacillus salivarius* BGH01	96	(Busarcevic and Dalgalarrondo, 2012)
5 (*T1 M1*)	59	Immunity	*Lactobacillus salivarius* BGH01	82	(Busarcevic and Dalgalarrondo, 2012)
6 (*Tb*)	69	Subunit B bacteriocin TmhB	*Blp1b, Lactobacillus salivarius* BGH01	100	(Busarcevic and Dalgalarrondo, 2012)
7 (*T1 M2*)	54	Immunity	*Lactobacillus salivarius* BGH01	100	(Busarcevic and Dalgalarrondo, 2012)
8 (*TIP*)	39	Induction peptide	*Lactobacillus salivarius* ATCC11741	79	(Harris et al., 2017)
9 (*TK*)	429	Histidine kinase	*Lactobacillus salivarius* ATCC11741	87	(Harris et al., 2017)
10 (*TR*)	264	Response regulator	*Lactobacillus salivarius* UCC118	99	(Flynn et al., 2002)
11 (*orf1*)	76	Putative protein	*Lactobacillus salivarius* ATCC11741	99	(Harris et al., 2017)
12 (*orf2*)	65	Putative protein	*Lactobacillus salivarius* ATCC11741	100	(Harris et al., 2017)
13 (*orf3*)	44	Putative protein	*Lactobacillus salivarius* ATCC11741	100	(Harris et al., 2017)
14 (*orf4*)	237	Putative protein	*Lactobacillus salivarius* ATCC11741	98	(Harris et al., 2017)
15 (*orf5*)	88	Putative protein	*Lactobacillus salivarius* CCUG47825	99	(Harris et al., 2017)
16 (*orf6*)	273	Integrase	*Lactobacillus salivarius* UCC118	99	(Flynn et al., 2002)

**Table 3:** Proteins encoded by the hypothetical two peptide-bacteriocin clusters of *Lactobacillus plantarum* C1, EML1 & SA3; and *L. paracasei* SA5.

Strains	ORF (gene)	Size (aa)	Function	Homologue (100%)	Reference
C1/EML1/SA3	1 (*W*)	228	CAAX protease self-immunity	*PlnW*, *Lactobacillus plantarum* C11	(Diep et al., 1996, 2009)
	2 (*V*)	226	CAAX protease self-immunity	*PlnV*, *Lactobacillus plantarum* C11	(Diep et al., 1996, 2009)
	3(*U*)	222	CAAX family putative protein	*PlnU*, *Lactobacillus plantarum* C11	(Diep et al., 1996, 2009)
	4 (*T*)	149	CAAX family putative protein	*PlnT, Lactobacillus plantarum* C11	(Diep et al., 1996, 2009)
	5 (*S*)	99	Putative protein	*PlnS*, *Lactobacillus plantarum* C11	(Diep et al., 1996, 2009)
	6 (*H*)	458	ABC-transporter accessory factor	*PlnH, Lactobacillus plantarum* C11	(Diep et al., 1996, 2009)
	7 (*G*)	716	ABC transporter	*PlnG, Lactobacillus plantarum* C11	(Diep et al., 1996, 2009)
	8 (*E*)	56	Plantaricin E	*PlnE*, *Lactobacillus plantarum* C11	(Diep et al., 1996, 2009)
	9 (*F*)	52	Plantaricin F	*PlnF, Lactobacillus plantarum* C11	(Diep et al., 1996, 2009)
SA5	1 (*A*)	77	Class IIb bacteriocin	*Lactobacillus casei* DPC6800	(Stefanovic et al., 2016)
	2 (*B*)	71	Class IIb bacteriocin	*Lactobacillus casei* UCD174	(Broadbent et al., 2012)
	3(*C*)	102	Putative protein	*Lactobacillus casei* W56	(Hochwind et al., 2012)
	4 (*AT*)	198	Acetyltransferase	*Lactobacillus casei* W56	(Hochwind et al., 2012)
	5 (*D*)	111	Immunity	*Lactobacillus casei* DPC6800	(Stefanovic et al., 2016)
	6 (*E*)	225	Metalloprotease	*Lactobacillus casei* W56	(Hochwind et al., 2012)
	7 (*F*)	110	Putative protein	*Lactobacillus casei* W56	(Hochwind et al., 2012)
	8 (*G*)	52	Putative membrane protein	*Lactobacillus casei* BL23	(Maze et al., 2010)

**Table 4:** Proteins encoded by the hypothetical bacteriolysin genes identified in the lactobacilli genomes.

Protein	Gene	Size (aa)	Conserved catalytic domain	Lactobacilli^a^	Homologue (100%)	Reference
Phage lysin	*acm*	245–772	M23 endopeptidase GH25-PlyB-like muramidase	C1, EML1, SA3 C2, C12, SA5	*Lactobacillus* sp. CBA3606 *L. paracasei* ATCC 25302 *L. salivarius* CECT 5713	NCBI Complete Genomes (Ward and Timmins, 1999) (Martin et al., 2006)
Lysozyme	*lyc*	309–921	GH25-LysA-like endolysin	C2, C12, SA5	*L. reuteri* TD1 *L. casei* 32G	(Leonard et al., 2014) (Aktas et al., 2015)
Autolysin 1	*lytA_1*	486	C39 endopeptidase	C2	*L. salivarius* cp400	(Mackenzie et al., 2014)
Autolysin 2	*lytA_2*	350–468	PGRP-family amidase	C2, C12, SA5	*L. salivarius* ACS-116-V-Col5a *L. casei* 32G	NCBI Complete Genomes (Aktas et al., 2015)
Amidase	*lytC*	282–350	MurNAc-LAA	C1, EML1, SA3 C2, C12, SA5	*Lactobacillus* sp. CBA3606 *Lactobacillus* sp. DS22_6	NCBI Complete Genomes
Toxin 1	*toxA_1*	125	Amidase	C2	*L. salivarius* cp400	(Mackenzie et al., 2014)
Toxin 2	*toxA_2*	523	M23 endopeptidase	C2, C12	*L. salivarius* cp400	(Mackenzie et al., 2014)

a *Lactobacilli strains in which the genes have been identified*.

### The Genes Encoding for the Two-Peptide Bacteriocins of *L. plantarum* and *L. casei* Over-Express in the Presence of Bacillus Calmette-Guerin Cells

**Figure 5:**
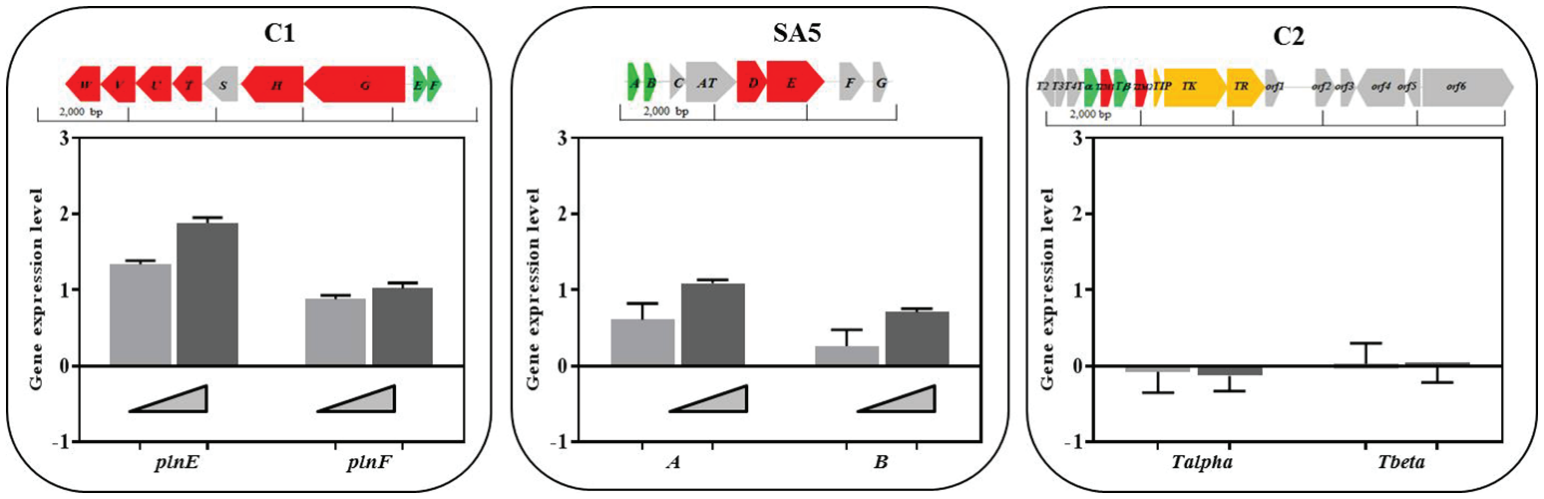
Class II bacteriocin gene clusters identified in the genome of the lactobacilli strains and level of expression of genes encoding for the hypothetical precursor bacteriocins in lactobacilli cultures exposed to increasing concentrations of BCG cells. The nomenclature for the bacteriocin clusters follows specific recommendations (Diep et al., 2009; O’Shea et al., 2011) and represents precursor bacteriocins (green), posttranslational modification enzymes (blue), transport/immunity proteins (red) and other hypothetical proteins (gray). C1. Two peptide-bacteriocin cluster of *L. plantarum* strains C1 (as a representative of all *L. plantarum* strains) and level of expression of its corresponding precursor bacteriocin genes *plnE* and *plnF* in cultures exposed to BCG cells at 10^6^ CFU/ml (light gray bars) and 10^7^ CFU/ml (dark gray bars). SA5. Two peptide-bacteriocin cluster of *L. casei* SA5 and level of expression of its corresponding precursor bacteriocin genes *A* and *B* in cultures exposed to increasing concentrations of BCG cells at 10^6^ CFU/ml (light gray bars) and 10^7^ CFU/ml (dark gray bars). C2. Single-peptide bacteriocin cluster of *L. salivarius* C2 and level of expression of its corresponding precursor bacteriocin genes *Tα* and *Tβ* in cultures exposed to BCG cells at 10^6^ CFU/ml (light gray bars) and 10^7^ CFU/ml (dark gray bars).

In order to determine whether the presence of BCG cells may regulate the level of expression of the identified class II bacteriocins we carried out an RT-PCR to quantify the amount of transcripts derived from the genes encoding for the hypothetical precursor bacteriocins in all *L. plantarum* strains, *L. salivarius* C2 and *L. casei* SA5 (indicated in green on top of Figure 5). The RNA was isolated from cultures of lactobacilli exposed to increasing concentrations of BCG cells during exponential growth. As illustrated in Figure 5, the level of expression of genes *plnE* and *plnF* was dependent on the amount of BCG cells used, with a very significant increase by comparison with *L. plantarum* cultures on their own. We observed similar results with *A/B* genes of *L. casei* SA5 although the increase was much lower. By contrast, the expression of genes *Tα/Tβ* in *L. salivarius* C2 was not affected by the presence of BCG cells (Figure 5). These data confirm not only the inducing effect of BCG cells on the anti-microbial activity displayed by *L. plantarum* and *L. casei* in co-cultures with BCG but also the possible role of two-peptide bacteriocins is such antimicrobial activity against BCG.

### Lactobacilli Influence Bacillus Calmette-Guerin Phagocytosis in a Species-Dependent Manner

**Figure 6:**
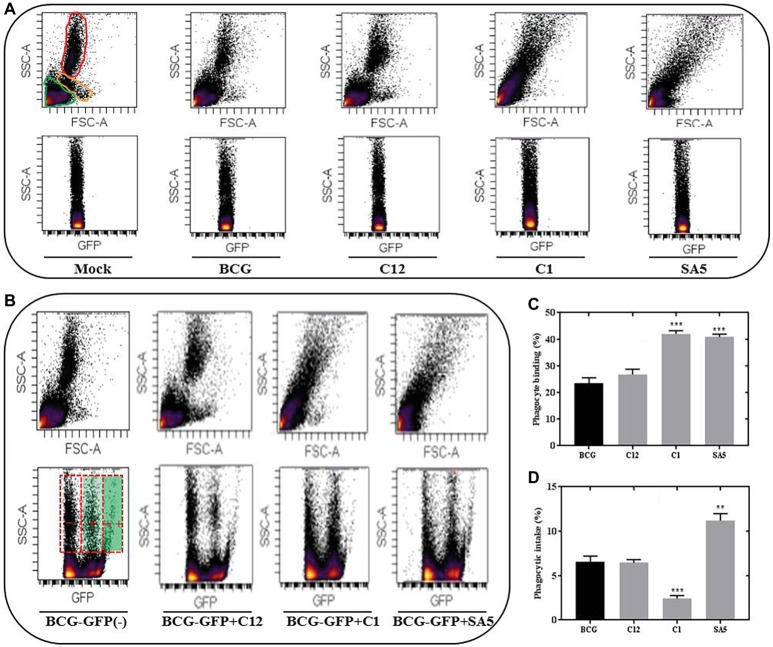
Lactobacilli interact with porcine blood cells, interfering with BCG phagocytosis in a species-dependent manner. **(A)** SSC/FSC plot areas representing porcine blood cells and their corresponding SSC/GFP areas when exposed to non-stimulated conditions (mock), BCG, *L. salivarius* C12, *L. plantarum* C1 and *L. casei* SA5. For the mock experimental conditions the blood cells are indicated in green (lymphocytes), orange (monocytes) and red (PMNs). **(B)** SSC/GFP plot areas illustrating the response of porcine blood cells to BCG-GFP alone (−) or in combination (+) with *L. salivarius* C12, *L. plantarum* C1 and *L. casei* SA5. The SSC/FSC areas for each of the conditions are included above and the GFP intensity recorded from phagocytes is indicated on the BCG-GFP experimental condition as negative (−), positive (+) and very positive (++). **(C)** Percentage of phagocytes that bind BCG when tested alone (black bar) or in combination (gray bars) with *L. salivarius* C12, *L. plantarum* C1 and *L. casei* SA5. Data are mean ± SD with Student’s *t*-test statistical analysis (***p < 0.001). **(D)** Percentage of phagocytes that phagocytize BCG when tested alone (black bar) or in combination (gray bars) with *L. salivarius* C12, *L. plantarum* C1 and *L. casei* SA5. Data are mean ± SD with Student’s *t*-test statistical analysis (**p < 0.01, ***p < 0.001).

Representative FSC-SSC plots indicating the presence of lymphocytes, monocytes and PMNs (neutrophils) in porcine blood are illustrated in Figure 6A. Unlike BCG and *L. salivarius*, the presence of *L. plantarum* and *L. casei* alters significantly the blood scatter profile, which complicated the identification of monocytes and neutrophils. Nevertheless, the different distribution of lymphocytes and phagocytes that we observed on the SCC-GFP plots under all bacterial conditions, let us distinguish between blood cells that are positive for BCG binding or BCG intake based on the GFP intensity. Lymphocytes and some phagocytes were found to be GFP positive but the highest GFP intensity was only observed in the phagocytic cells (Figure 6B). *L. salivarius* (C12) had no significant effect on the BCG binding (Figure 6C); neither on the phagocytic response to BCG (Figure 6D). However, the percentage of phagocytes positive for BCG binding and BCG intake changed significantly in the presence of *L. plantarum* (C1) and *L. casei* (SA5) Although both lactobacilli increased the binding between BCG and phagocytes (Figure 6C) their effect on BCG phagocytosis was completely different (Figure 6D). *L. plantarum* reduced the BCG intake, whereas *L. casei* caused the opposite effect.

## Discussion

To the best of our knowledge, this is the second study reporting the isolation of *Lactobacillus* spp. from wild boar. The only previous publication is focused on the antibiotic susceptibility of lactobacilli isolates and the authors found *Lactobacillus* species are different from this study, including *L. mucosae*, *L. reuteri*, *L murinus,* and *L. fermentus* (Klose et al., 2014). In our study we describe, for the first time, that wild boar carry *Lactobacillus* spp. such as *L. plantarum*, *L. salivarius* and *L. casei* that exert antimycobacterial activity when they compete against *M. bovis* BCG in co-cultures. Our results are consistent with previous studies that found that BCG and *M. bovis* are inhibited by lactobacilli isolated from badger feces or present in fermented milk products (Mariam, 2009; Macuamule et al., 2016; Stedman et al., 2018). We initially suspected that the antimicrobial activity that our lactobacilli isolates display against BCG was due to acidic pH but low pH was insufficient to induce a decrease in the survival of BCG when grown as a mono-culture as previously reported (de la Rua-Domenech, 2006; Stedman et al., 2018). In fact, *M. bovis* naturally resist acidic pH due to their the ability to adopt intracellular homeostasis (Rao et al., 2001).

Overall the lactobacilli isolates that we have isolated in this study seem to produce metabolites that display a very significant antimicrobial activity against BCG, but only at low pH, suggesting a synergistic effect at acidity conditions. All our isolates contain genes associated with the synthesis of lactate and acetate, which inhibit active transport in other bacteria by causing interference on the membrane potential; as well as ethanol, CO_2_ and hydrogen peroxide, additional antimicrobial compounds that may prevent bacterial growth by creating a hostile environment (Pessione, 2012). It has been well-documented that the antibacterial activity of lactobacilli is multifactorial, including lowering of the pH with simultaneous production of lactic acid and of non-lactic acid metabolites (Fayol-Messaoudi et al., 2005). These antimicrobial metabolites could even act in synergy since lactic acid permeabilizes the outer membrane of bacteria, allowing the non-lactic acid metabolites to enter the bacterial cells (Alakomi et al., 2000). This potential synergistic effect between acidic pH and antimicrobial metabolites was further supported by the lack of antimycobacterial activity that we observed at neutral pH. All the lactobacilli mono-cultures provided with metabolites that were incapable of inhibiting the growth of BCG at pH 7.

The reasons why metabolites from the lactobacilli mono-cultures display no activity against BCG at pH7 could also lie on the fact that some bacteriocins are only fully functional at acidic conditions. The genome of all the lactobacilli isolates showed genes related to the biosynthesis of bacteriolytic class III bacteriocins known as bacteriolysins, especially in *L. salivarius*. Bacteriolysins are enzymes that have been adapted to the host habitat of acidification, with an optimal bactericidal activity that ranges between pH values of 4 and 6 (Sable and Lortal, 1995; Ribelles et al., 2012). This could also explain the absence of antimicrobial activity that we recorded against BCG using metabolites from all the *L. salivarius* co-cultures at pH7. However, neutral pH had no influence on the antimycobacterial activity observed with the metabolites derived from co-cultures of BCG with *L. plantarum* and *L. casei*. This remarkable observation suggests that, first, such antimycobacterial activity is dependent on the lactobacilli species and is not associated with low pH; and, second, BCG triggers its own bactericidal effect. Interestingly, we found that the strains of *L. plantarum* and *L. casei* carry gene clusters associated with the production of two-peptide bacteriocins, also known as class IIb bacteriocins, which consist of two different peptides that confer optimal antimicrobial activity if both peptides are present in equal amounts (Nissen-Meyer et al., 2010). Two-peptide bacteriocins often display an antibacterial activity that is very stable at a very broad range of pH (1–11); and the genes that encode for the two peptides are next to each other in the same operon where other genes associated with processing, transport and immunity are located.

The genes that encode for the two-peptide bacteriocin that we identified in the genome of the three *L. plantarum* strains are identical to the two genes of operon *plnEF*. Genes *plnE* and *plnF* allow the expression of two peptides, plantaricins E and F, that interact together to form a helix-helix structure that binds to a specific membrane protein of the target bacteria, presumably a transporter of the APC superfamily, leading to membrane leakage and cell death (Ekblad et al., 2016; Oppegard et al., 2016). The remainder of the genes that we detected in the *L. plantarum* genomes are identical to operon *plnWVUTSHG*, which are thought to be involved in transport, processing and self-protection (Diep et al., 2009). The operons *plnEF* and *plnWVUTSHG* are frequently found in many *L. plantarum* strains, along with other two-peptide bacteriocin clusters such as plantaricin JK, although it has been reported to be on their own in certain isolates (Saenz et al., 2009; Tai et al., 2015). Furthermore, we detected a gene cluster involved in the hypothetical synthesis of a novel two-peptide bacteriocin in *L. casei*. The gene cluster is composed of two genes that encode for 2 bacteriocins that show certain similarity with thermophilin A (Ward and Somkuti, 1995) and carnocin CP51 (Herbin et al., 1997), followed by a galactoside O-acetyltransferase gene prior to two additional genes involved in immunity and transport. Carnocin CP51 is expressed with the complementary peptide carnocin CP52 in *Carnobacterium* and thermophilin A glycosylates as it occurs with some plantaricins (Diep et al., 2009).

As a very interesting fact we observed that the two gene operons that encode for the two-peptide bacteriocins in *L. plantarum* and *L. casei* over-express in the presence of BCG cells in a dose-dependent manner. These results could explain why metabolites that derive from co-cultures of BCG with *L. plantarum* and *L. casei*, show anti-mycobacterial activity. Neither monocultures of these two *Lactobacillus* spp. or their metabolites after propagation in supernatants containing BCG metabolites displayed any antimycobacterial activity. In fact, the regulation of plantaricin production had been previously described in co-cultures of *L. plantarum* (Maldonado et al., 2004b; Maldonado-Barragan et al., 2013). In agreement with our transcriptional data, a previous study has reported that operon of the two-peptide bacteriocin plantaricin NC8 of *L. plantarum* NC8 is up-regulated in broth only after cultivation with other gram-positive bacteria or the addition of heat-killed cells from some of the inducing bacteria (Maldonado et al., 2004a). Whether two-peptide bacteriocins such as plantaricins are involved in the antimycobacterial activity that we have observed in this study is a question that remains unanswered. Based on our results it is too early to speculate about the possible role of plantaricins as antimicrobials against mycobacteria; mainly because of their relatively narrow inhibitory spectra, which includes other species of lactobacilli as well as gram-positive closely related bacteria such as *Pediococcus* (Diep et al., 2009). However, this potential anti-mycobacterial role is worthy of further investigation due to the promising contribution of plantaricins to probiotic functionality when colonizing the gut (Maldonado-Barragan et al., 2013) and the fact that class II bacteriocins have antimicrobial activity against *M. tuberculosis* (Sosunov et al., 2007).

Unlike the two-peptide bacteriocins, the two identified single-peptide bacteriocins of *L. salivarius* showed no transcriptional up-regulation in the presence of BCG cells, which is in accordance with the evidence that metabolites from *L. salivarius* have no influence on the survival of BCG at pH7. The two bacteriocins that we found in the genome of *L. salivarius* show similarities with multiple class IIb bacteriocins previously described in other *L. salivarius* strains (O’Shea et al., 2011) but also with bacteriocins blp1α/blp1β of *L. salivarius* BGHO1 (Busarcevic and Dalgalarrondo, 2012) and ThmA/ThmB (Thermophilin 13) of *Streptococcus thermophilus* (Marciset et al., 1997). Our two *L. salivarius* bacteriocins contain the double-glycine leader sequence of class II bacteriocins but, in contrast to two-peptide bacteriocins (class IIb), their operons carry immunity genes for each of the bacteriocin precursors. As these bacteriocins also lack the YGNGV-C consensus sequence of class IIa bacteriocins the bacteriocin cluster was classified as a single-peptide bacteriocin (class IId). The evidence that bacteriocins blp1α/blp1β are produced only after culturing in a very chemically defined medium and that bacteriocins ThmA/ThmB function without a bacterial membrane receptor may also explain the absence of antimycobacterial activity observed with the *L. salivarius* metabolites at neutral pH.

Another important aspect that is worth mentioning is the fact that nutrient deprivation caused by lactobacilli metabolism seems to have no influence on the survival of BCG. The viability of BCG that we have recorded following incubation in pH7-adjusted supernatants from lactobacilli cultures, remains unaffected. In addition, lactobacilli grow well in the presence of BCG metabolites, suggesting that the metabolic pathways of *Lactobacillus* and *M. bovis* have no cross-interference. Lactobacilli, as many other lactic acid bacteria, have a relatively simple metabolism that converts sugars to pyruvate *via* the glycolytic pathway, generating energy (Papagianni, 2012b), whereas mycobacteria shows a very complex, but flexible, central carbon metabolism that generates energy from glycolysis, gluconeogenesis, the pentose phosphate pathway, and the TCA pathway depending on the resources available (Cumming and Steyn, 2015). These metabolic facts, together with the evidence that acidic pH has no *in vitro* anti-mycobacterial effect, reinforce our hypothesis that lactobacilli exert antimicrobial activity against BCG through synergistic mechanisms that include combination of acidity with different metabolites such as organic acids, hydrogen peroxide, ethanol and bacteriocins.

We finally determined the phagocytic response to BCG in the presence of lactobacilli. Isolates of *L. plantarum* and *L. casei* had a significantly opposite effect on BCG phagocytosis. *L. plantarum* decreased the BCG intake, whereas *L. casei* increased the phagocytic response, suggesting a completely different interaction between these two species and the phagocytes. Therefore, we believe that *L. plantarum* and *L. casei* may be recognized by different phagocytic receptors that, to a greater or lesser extent, could also be involved in the recognition of BCG. Phagocyte internalization is clearly beneficial to the survival of *M. tuberculosis* complex species, which may enter macrophages *via* different receptor molecules, including complement receptors involved in the classical and alternative pathways such as C1 and C-type lectin receptors (Pieters, 2008; Marakalala et al., 2018). In this respect, very recent studies have described that lactobacilli are able to express adhesins that bind to C-type lectin receptors (Bene et al., 2017). Interestingly, the genomes of our *L. plantarum* isolates have revealed the presence of *cna*, a gene that encodes a collagen adhesin (*cna*) and that is absent in the *L. casei* genome. As this collagen adhesive protein has also been reported to block the C1-dependent complement activation (Kang et al., 2013), the antagonism that *L. plantarum* shows against BCG intake could be caused by competition not only for lectin receptors but also for the classical complement activation pathway.

The reason why *L. casei* increases BCG phagocytosis is intriguing and could be due to many different factors including the expression of *spaD*, a fimbrial protein gene that we have identified in its genome. This protein shows a very high similarity with the backbone-pilin subunit spaD, and fimbriae (or pili) of lactobacilli have been reported to facilitate phagocytosis in macrophages *via* integrin CD11b/CD18 (Vargas Garcia et al., 2015), also known as complement receptor 3 (CR3). On the other hand, CR3 may act as a negative regulator of C-type lectin receptors (Zhang et al., 2018) and seems not to alter the progression of *M. tuberculosis* infection (Hu et al., 2000). Therefore, fimbriae could promote BCG macrophage uptake through indirect positive collaboration with main bTB phagocytic receptors such as lectins. Phagocytic receptors that could have no significant influence on BCG intake might include sensors for sialic acid recognition as both *L. plantarum* and *casei* contain the serin-rich adhesin gene *sraP* in their corresponding genomes (Kline et al., 2009).

Isolates of *L. salivarius* seem to have no interaction with phagocytes as they caused no significant effects on the BCG intake, neither on the BCG binding. By contrast, *L. plantarum* and *L. casei* clearly stimulated phagocytosis, as explained above, as well as BCG binding. These two lactobacilli species also altered significantly the porcine blood profile. Lactobacilli are known to activate macrophages *via* Toll-like Receptors (TLR) and our 3 *Lactobacillus* species contain the genes *ltaS1* and *ltaS2* involved in the synthesis of lipoteichoic acid (LTA), a major constituent of the cell wall that acts as a TLR2 stimulator (Sengupta et al., 2013). However, *L. plantarum* and *L. casei* also harbor genes encoding other components of the cell wall and the cytoplasmic membrane that are absent in *L. salivarius*, such as the *L. casei* spaD-like fimbrial protein and the genes *tagBFGH* associated with the biosynthesis of cell wall teichoic acids (WTA) in *L. plantarum*. Fimbriae and WTA are capable to activate macrophages *via* TLR5 and 2, respectively (Ganguli et al., 2015; Hevia et al., 2015; Yu et al., 2015) and, under our experimental conditions, their contribution towards phagocyte stimulation could be much more significant than LTA. TLR activation may collaborate with phagocytic receptors with subsequent implications on the uptake process (Gordon, 2016).

## Conclusions

We started from the premise that commensal bacteria such as lactobacilli could play an important, and hitherto underappreciated role in the outcome of bTB in wild boar. In our study we have found that lactobacilli isolated from wild boar influence the viability of *M. bovis* BCG and modify its phagocytic intake in a species-dependent manner. In particular, isolates of *L. plantarum* showed antimicrobial and immunomodulatory properties that could antagonize *M. bovis* survival. The genome of these isolates revealed the presence of two-peptide bacteriocins and a collagen adhesive protein that could act as antimycobacterials and innate immunomodulators; a very important question that remains to be elucidated. Our preliminary results have been generated from reductionist *in vitro* assays, but bring positive prospects with regards to the potential use of lactobacilli as an additional competitive pressure to control bTB in wild boar. In this respect, *in vitro* and *in vivo* work with pathogenic *M. bovis* will be needed to further explore this possibility. Oral administration of lactobacilli with antimycobacterial activity could reduce the gut burden of *M. bovis*, thus reducing the risk of transmission of bTB between domestic and wild animals, provided that fecal shedding is one of the main excretion routes of mycobacteria (Santos et al., 2015).

## Ethics Statement

All experiments included in this study involve no animals and the sampling procedures were performed under safe and protective legal regulations. Fecal samples were collected from 30 wild boar of which 20 were live animals and the remainder 10 dead after being hunted in legal recreational events on which we did not take any participation at all. The samples from the 20 live animals were taken from young wild boars aged between 4 and 8 months using feeding-traps under safe and protective legal regulations in accordance with the required animal husbandry procedures that meet the guidelines set by the appropriate Regional College of Veterinary Surgeons. The samples from the 10 dead animals were randomly collected among others aging between 1 and 3 years old by registered veterinary surgeons at official certified abattoirs. The porcine blood was collected in heparinized tubes from healthy pigs at the Pirbright Institute (UK), where all animal procedures are covered by a license issued by the UK Home Office under the Animal (Scientific Procedures) Act1986.

## Author Contributions

MB and TC planned, performed experiments, and edited the manuscript. FM designed experiments, aided in data analysis, and aided in preparing and editing the manuscript. WG-J and PF-L conducted the sampling and edited the manuscript. JR and DR designed experiments and aided in preparing and editing the manuscript. JG-M designed and performed experiments, aided in data analysis, and prepared and edited the manuscript. All authors read and approved the final manuscript.

### Conflict of Interest Statement

The authors declare that the research was conducted in the absence of any commercial or financial relationships that could be construed as a potential conflict of interest.

## Footnotes

^1^https://github.com/tseemann/shovill
