# Supplementary material for: Lactobacilli Isolated From Wild Boar (Sus scrofa) Antagonize Mycobacterium bovis Bacille Calmette-Guerin (BCG) in a Species-Dependent Manner
Source: Front Microbiol. 2019 Jul 30;10:1663. doi: 10.3389/fmicb.2019.01663 (PMC6683848; doi:10.3389/fmicb.2019.01663)
Supplement: Supplementary file 1 [file Data_Sheet_1.pdf]

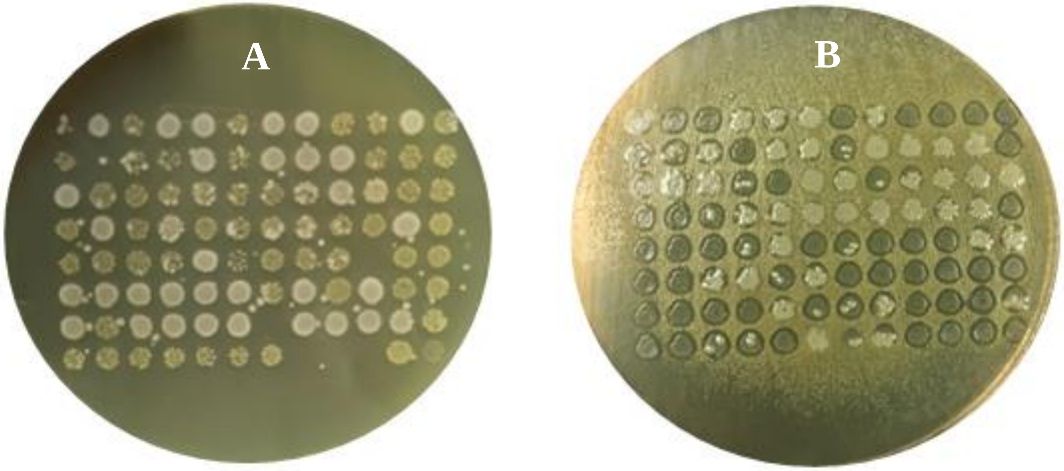

Fig. S1. A representative experiment illustrating the selection of lactic acid bacteria (LAB) cultures with anti-mycobacterial activity. LAB cultures obtained in 96-well plates are replica plated twice; first, onto a Tryptone Soya Agar (TSA) plate (A); and second, onto another TSA plate that has previously been swabbed with *Mycobacterium smegmatis* (B). After incubation at 37°C for 48h, cultures showing halos of antimicrobial activity against *M. smegmatis* on the second TSA plate (B) are selected from the first TSA plate (A) for further LAB colony isolation and identification.

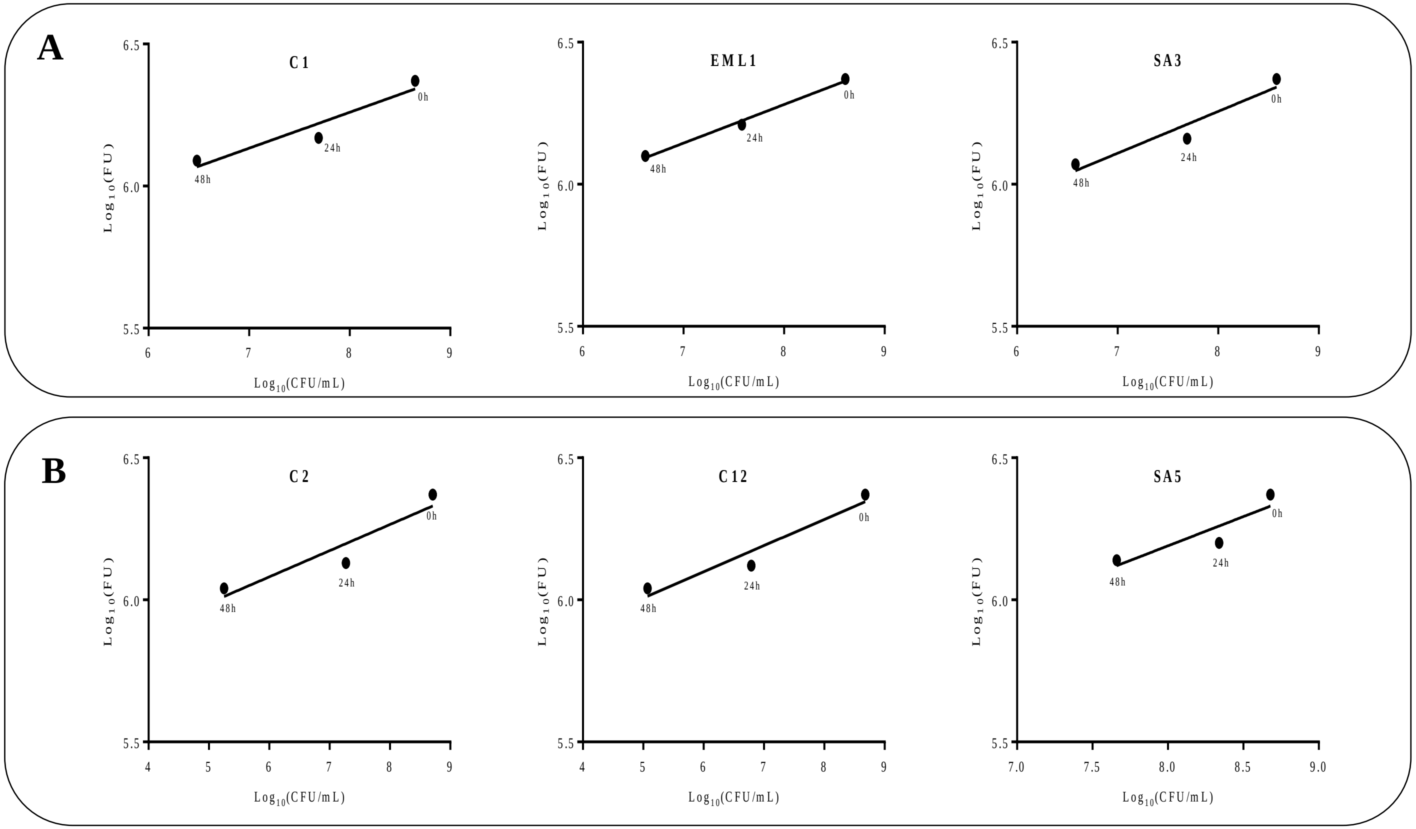

Fig. S2. Correlation between mean values of $\log_{10}$(CFU/mL) and $\log_{10}$(FU) of BCG in co-cultures with *L. plantarum* isolates (A) and isolates of *L. salivarius* and *L. casei* (B) after 48 hours (h) of incubation in MH broth with OADC (10%), Tween 80 (0.1%) and glycerol (0.2%). The graphs show a positive goodness of fit between $\log_{10}$ (CFU/mL) and $\log_{10}$(FU) ($R^2 > 0.9$).

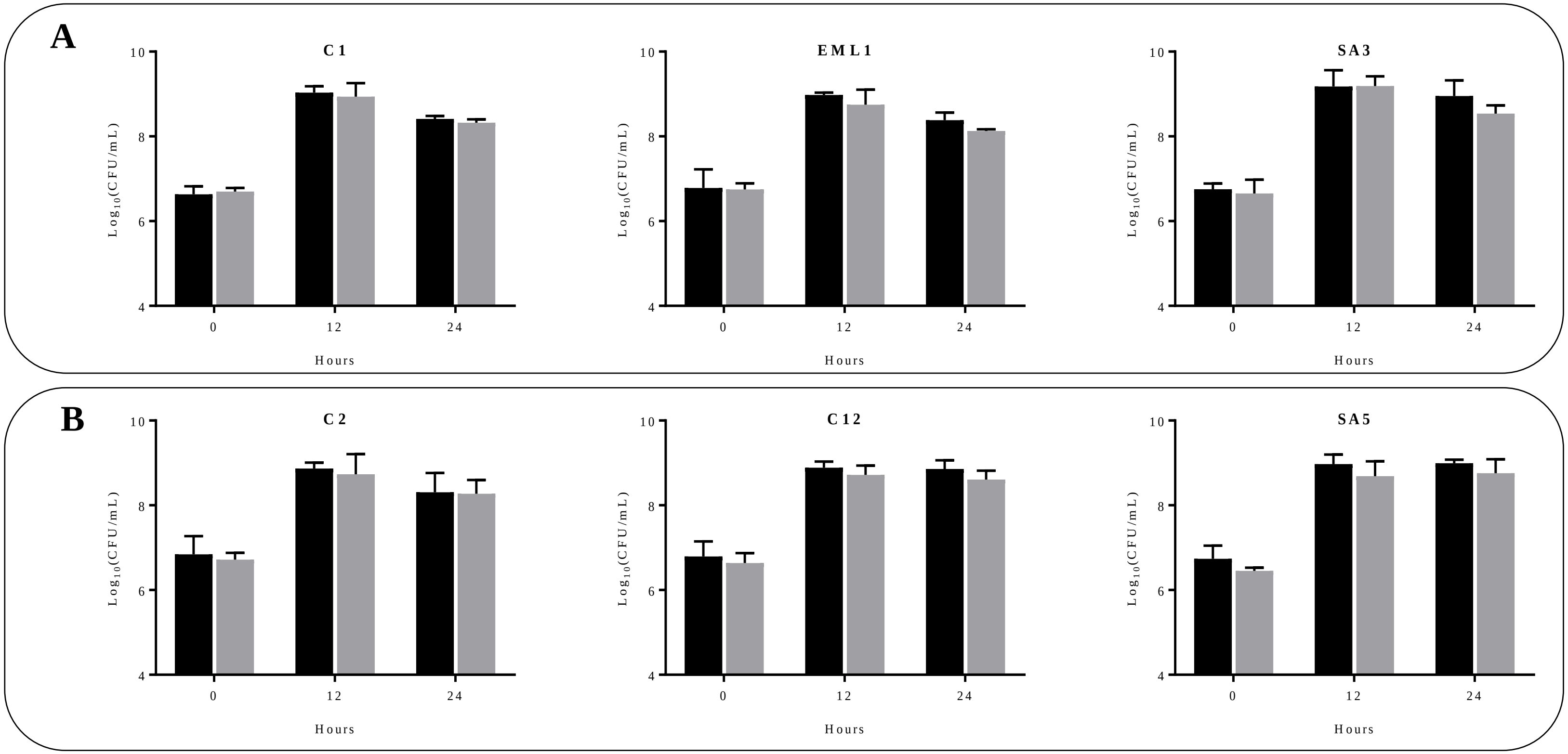

Fig. S3. Bacterial counts of *L. plantarum* isolates (A) and isolates of *L. salivarius* and *L. casei* (B) as mono-cultures (black bars) and co-cultures with BCG (grey bars) after an incubation of 0, 24 and 48h in MH broth with OADC (10%), Tween 80 (0.1%) and glycerol (0.2%). Bacterial counts are indicated as $\log_{10}$(CFU/mL).

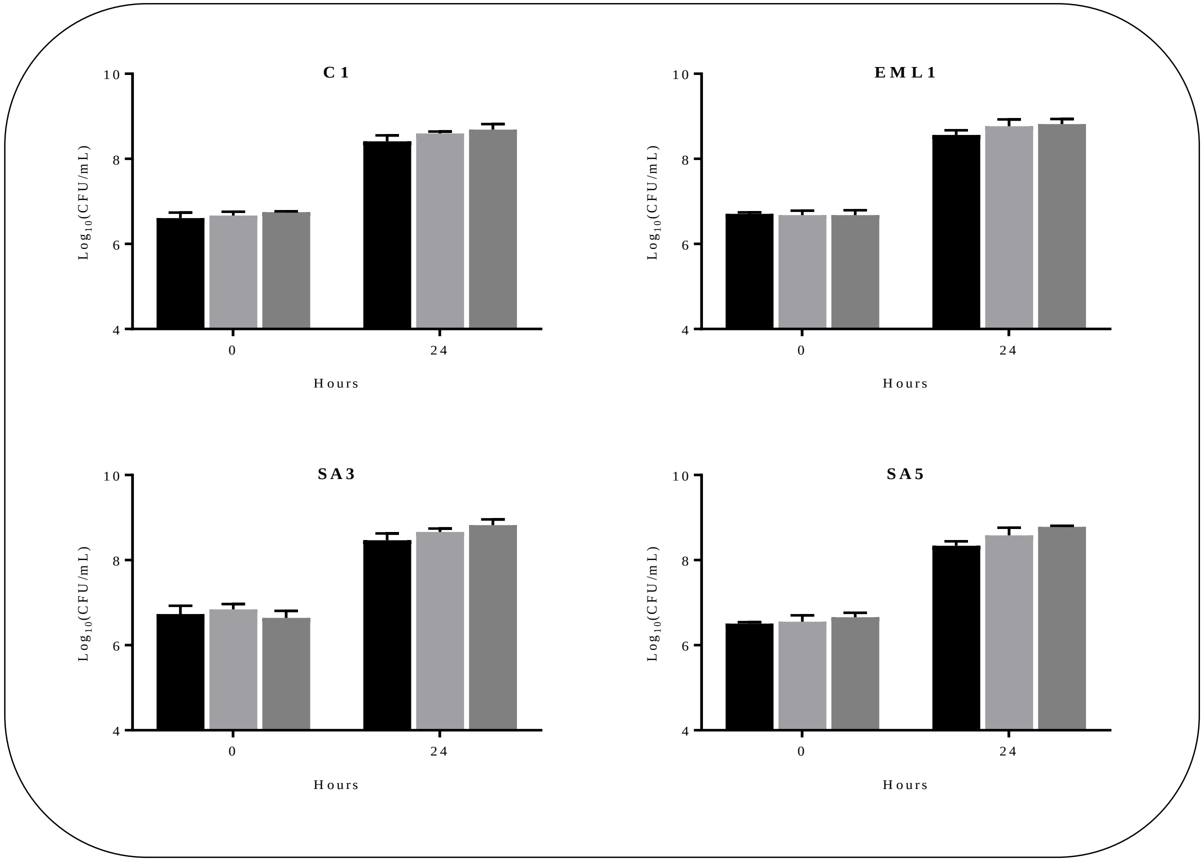

Fig. S4. Bacterial counts from cultures of *L. plantarum* isolates C1, EML1 & SA3 and *L. casei* SA5 after an incubation of 0 and 24h in: (i) supernatants from a 5 day-old BCG culture (black bars); (ii) supernatants from a 5 day-old BCG culture supplemented with MH broth with OADC (10%), Tween 80 (0.1%) and glycerol (0.2%) at a ratio of 1:1 (dark grey bars); and (iii) MH broth with OADC (10%), Tween 80 (0.1%) and glycerol (0.2%) (light grey bars). Bacterial counts are indicated as log_{10}(CFU/mL).

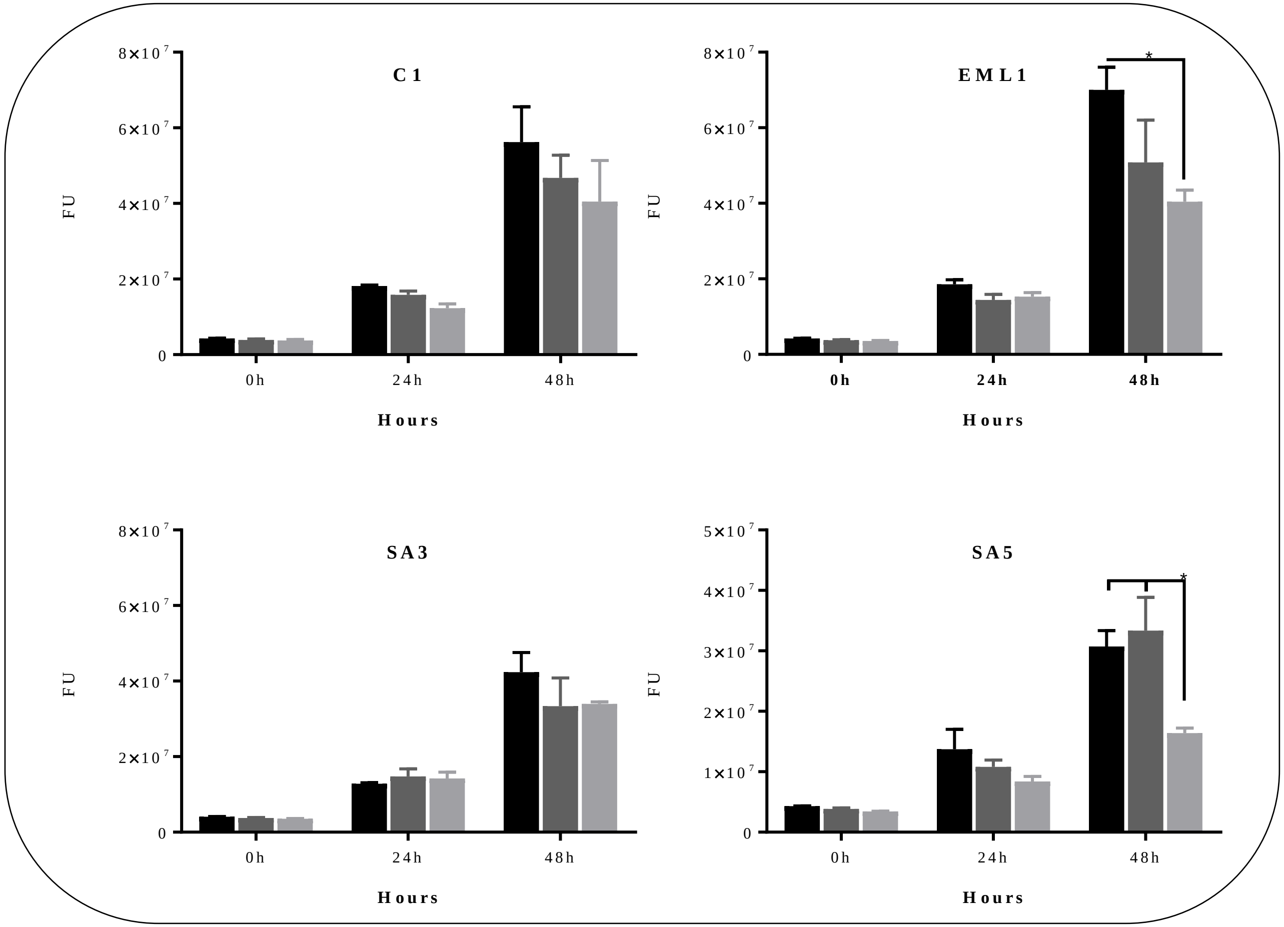

Fig. S5. Survival rate of BCG after 48 hours (h) of incubation in cell-free supernatants from cultures of *L. plantarum* C1, EML1 & SA3 and *L. casei* SA5 that were propagated in: (i) supernatants from a 5 day-old BCG culture (black bars); (ii) supernatants from a 5 day-old BCG culture supplemented with MH broth with OADC (10%), Tween 80 (0.1%) and glycerol (0.2%) at a ratio of 1:1 (dark grey bars); and (iii) MH broth with OADC (10%), Tween 80 (0.1%) and glycerol (0.2%) (light grey bars). The BCG survival rate was monitored as GFP expression and data are mean ± SD with statistical analysis (Student's *t*-test, *$p<0.05$) and are representative of 3 biological replicates each.
